# Sterols from the red algae, *Gracilaria salicornia* and *Hypnea flagelliformis*, from Persian Gulf

**DOI:** 10.4103/0973-1296.80663

**Published:** 2011

**Authors:** Masoumeh Nasir, Soodabeh Saeidnia, Ali Mashinchian-Moradi, Ahmad R. Gohari

**Affiliations:** *Department of Marine Science and Technology, Science and Research Branch, Islamic Azad University, Tehran, Iran*; 1 *Medicinal Plants Research Center, Faculty of Pharmacy, Tehran University of Medical Sciences, Tehran, Iran*

**Keywords:** *Gracilaria salicornia*, *Hypnea flagelliformis*, sterols, (22*E*)-cholesta-5, 22-dien-3β-ol-7-one

## Abstract

**Context:**

Two of the important algae from Persian Gulf are *Gracilaria salicornia* and *Hypnea flageliformis* (Rhodophyta). Antibacterial, antifungal, and cytotoxic effects of the mentioned algae have been presented in the previous studies.

**Aim:**

In this study, the isolation and structural elucidation of the sterols from these algae are reported.

**Materials and Methods:**

The separation and purification of the compounds were carried out with silica gel, sephadex LH_20_ column chromatography (CC) and HPLC to obtain six pure compounds **1-6**. The structural elucidation of the constituents was based on the data obtained from H-NMR,^13^C-NMR, HMBC, HSQC, DEPT, and EI-MS.

**Results:**

The isolated compounds from *G. salicornia* were identified as 22-dehydrocholesterol (1), cholesterol (2), oleic acid (3), and stigmasterol (4), and the isolated constituents from *H. flagelliformis* were identified as 22-dehydrocholesterol (1), cholesterol (2), oleic acid (3), cholesterol oleate (5), and (22*E*)-cholesta-5,22-dien-3β-ol-7-one (6) based on the spectral data compared to those reported in the literature.

**Conclusion:**

Red algae are enriched with cholesterol polysaccharides. We first reported the presence of cholesteryl oleate and (22*E*)-cholesta-5,22-dien-3β-ol-7-one in *H. flagelliformis*.

## INTRODUCTION

Algae are the large and diverse organisms among the marines, from which many secondary metabolites have been isolated. Some of these compounds possess biological and pharmacological activities.[1–3] Cytotoxic gracilarioside and gracilamides from *Gracilaria* species, a porcine pancreas elastase (PPE) inhibitor, diketosteroid, from *Hypnea musciformis*, and a new lectinof H. *cervicornis* have already been reported.[2,5] Bioactive sterols of *Cladophora rivularis,* anticoagulant sulfated polysaccharidesof Dictyota menstrualis, and an anti-HSV-1 agent from Cystoseira myrica have been reported.[6,8] Here we report the phytochemical analysis of G. salicornia and H. flagelliformis, which were previously reported for antibacterial and cytotoxic effects, leading to finding their main sterols.[9]

## MATERIALS AND METHODS

### Algae material

Red algae were collected from northern coastal areas of Persian Gulf near to Bandare-Abbas city in July (2007) and identified as *Gracilaria salicornia* (C. Agardh) E. Y. Dawson and *Hypnea flagelliformis* Greville ex J. Agardh by Dr. Jelveh Sohrabipour. The voucher specimens (*G. salicornia*: 53-22R, and *H. flagelliformis*: 52-21R) are deposited at the Herbarium of Agriculture and Natural Resources Research Center of Hormozgan, Bandar Abbass, Iran.

### Experimental

^1^ H- and ^13^C-NMR spectra were measured on a Brucker Avance 500 DRX (500 MHz for^1^ H and 125 MHz for^13^C) spectrometer (Germany) with tetramethylsilane as an internal standard, and chemical shifts are given in δ (ppm). EI-MS data were recorded on an Agilent Technology (HP) instrument (USA) with the 5973 network mass selective detector (MS model). Silica gel 60F_254_ precoated plates (Merck, Germany) were used for TLC. The spots were detected by spraying anisaldehyde-H_2_ SO_4_ reagent followed by heating. Sephadex LH_20_ was from Fluka (Switzerland). HPLC was used on a Knauer model with Vertex (Knauer, Germany) column C18 (250 × 20 mm ID). Detector was PDA (UV spectra were collected across the range of 200-900 nm) and injection volume was 2 ml.

### Extractions of the marine algae

Marine algae were dried carefully and reduced to small pieces. The dried powder of both algae (1.5 kg) was extracted with chloroform: methanol (3:1) by percolation (72 h) at room temperature. The solvents were then evaporated under reduced pressure to obtain the concentrated extracts, and dried under vacuum in order to give a dried powder of the extracts of *H. flagelliformis* and *G. salicornia* (13 g and 15 g, respectively). The dried extract was dissolved in ethyl acetate and decanted three times with distilled water. The ethyl acetate (supernatant) and aqueous parts were dried to gain 3.2- and 9.8-g extracts of *H. flagelliformis*. The ethyl acetate (supernatant) and aqueous extracts of *G. salicornia* were found to be 4 and 11 g.

### Isolation of the main constituents

#### G. salicornia

The ethyl acetate extract (4 g) was submitted to the silica gel column chromatography (CC) with hexane, chloroform, CHCl_3_:AcOEt (1:1), AcOEt, and MeOH consequently, to obtain six fractions (A-F). Fraction E (400 mg) was subjected to silica gel CC, with CHCl_3_ and CHCl_3_:AcOEt (5:5) to give five fractions (E_1_ -E_5_ ). Fractions E_2_ and E_3_ were pure compounds 1 (12 mg) and 2 (19 mg). Fraction E_5_ (150 mg) was purified on sephadex LH_20_ with MeOH as an eluent to obtain four fractions (E_51_ -E_54_ ). Fraction E_53_ was the pure compound 3 (7 mg). Compound 4 (12 mg) was obtained from silica gel CC, with CHCl_3_:AcOEt (8:2), from fraction E_54_ (66 mg).

#### H. flagelliformis

The ethyl acetate extract (3.2 g) was submitted to the silica gel CC with hexane, chloroform, CHCl_3_:AcOEt (1:1), AcOEt, and MeOH consequently, to obtain 10 fractions (A-J). The fraction E (368 mg) was subjected to silica gel CC, with CHCl_3_ and CHCl_3_:AcOEt (5:5) to give eight fractions (E_1_ -E_8_ ). Fractions E_6_ and E_7_ were pure compounds 1 (20 mg) and 2 (13 mg). Fraction E_8_ was purified on ephadex LH_20_ with MeOH as an eluent to obtain four fractions (E_81_ -E_84_ ). Fraction E_83_ was submitted to HPLC. Gradient elution (flow rate, 4 ml/min) is followed: Solvent system A was from 25% MeOH (initial state) to 10% MeOH (final state) during 40 min and solvent system B was from 10% MeOH to 100% H_2_ O during 20 min. Nine fractions were obtained using HPLC (E_831_ -E_839_ ). Fractions E_834_ and E_835_ were the pure compounds 3 (3 mg) and 5 (15 mg), respectively. Fraction H (208 mg) was subjected to sephadex LH_20_ with MeOH as an eluent to obtain five fractions (H_1_ -H_5_ ). Fraction H_3_ (83 mg) was submitted to HPLC. Gradient elution (flow rate, 4 ml/min) is followed: Solvent system A was from 75% MeOH to 90% MeOH during 40 min and solvent system B was from 90% MeOH to 100% MeOH during 20 min. Five fractions were obtained using HPLC (H_31_ -H_35_ ). Fraction H_33_ was the pure compound **6** (8 mg).

## RESULTS

**Figure 1:**
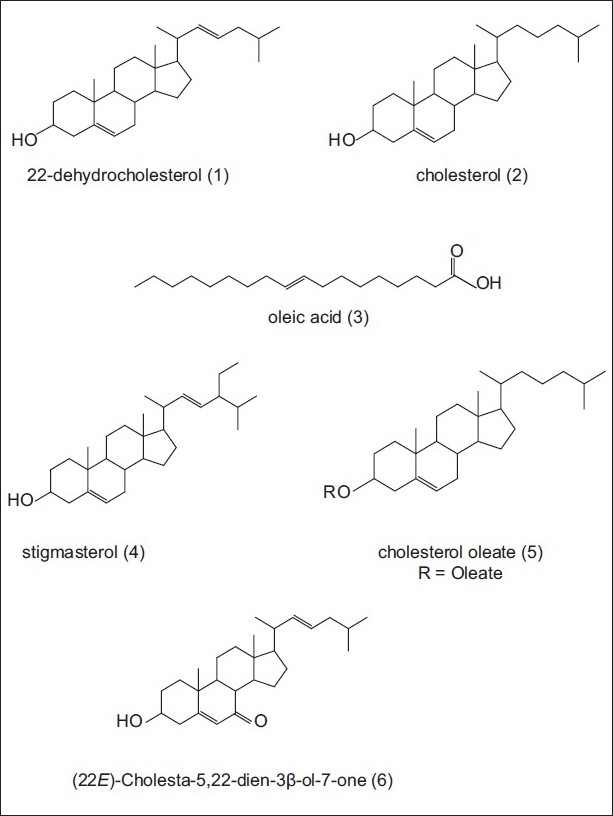
Structures of the isolated sterols from *Gracilaria salicornia* and *Hypnea flagelliformis*

Ethyl acetate extracts of two marine algae (Rhodophyta), *G. salicornia* and *H. flagelliformis*, were analyzed in order to isolate the sterol components. The separation and purification of the compounds were carried with silica gel CC and HPLC to obtain six pure compounds 1-6. The structural elucidation of the constituents was based on the data obtained from H-NMR,^13^C-NMR, HMBC, HSQC, DEPT, and EI-MS. The separated compounds from *G. salicornia* were identified as 22-dehydrocholesterol (1), cholesterol (2), oleic acid (3), and stigmasterol (4) based on the spectral data compared to those reported in the literature. The structures of isolated constituents from *H. flagelliformis* were elucidated as 22-dehydrocholesterol (1), cholesterol (2), oleic acid (3) cholesterol oleate (5), and (22*E*)-cholesta-5,22-dien-3β-ol-7-one (6) [Figure 1].[10–13]^13^C-NMR data of the sterols are summarized in Table 1.

**Table 1:** ^13^C -NMR data (δ ppm) of the sterols 1, 2, 4, 5, and 6, isolated from *Gracilaria salicornia* and *Hypnea flagelliformis*

No.	1	2	4	5	6
1	37.2	37.2	37.3	37.2	36.3
2	31.6	31.6	31.7	31.6	31.2
3	71.8	71.8	71.8	71.8	70.5
4	42.2	42.2	42.2	42.2	41.8
5	140.7	140.7	140.8	140.7	165
6	121.7	121.7	121.7	121.7	126.1
7	31.9	31.9	31.9	31.9	202.2
8	31.9	31.9	31.9	31.9	45.4
9	50.2	50.2	50.2	50.2	50
10	36.4	36.4	36.4	36.4	38.3
11	21.1	21.1	21.1	21.1	21.2
12	39.7	39.8	39.7	39.7	38.6
13	42.2	42.3	42.2	42.2	43
14	56.8	56.8	56.9	56.8	51.2
15	24.2	24.2	24.4	24.2	26.3
16	28.2	28.2	28.9	28.2	28.8
17	55.9	56.1	56	56.1	54.6
18	11.8	12	12	12	12.2
19	19.3	19.3	19.4	19.3	17.3
20	40.1	35.8	40.5	35.8	39.9
21	20.8	18.7	21.2	18.7	21
22	128.1	36.2	138.3	36.2	137.9
23	126.2	23.8	129.3	23.8	126.4
24	41.9	39.5	51.6	39.5	41.9
25	28.6	28	31.9	28	28.5
26	22.3	22.7	19	22.7	22.3
27	22.2	22.6	21.1	22.6	22.3
28	-	-	25.4	-	-
29	-	-	12.2	-	-

## DISCUSSION

Cholesterol and 22-dehydrocholesterol are found in these algae as two of the main sterols. This is in agreement with the literature which stated that most of the red algae (Rhodophyta) contain primarily cholesterol, although several species contain large amounts of desmosterol, and one species contains primarily 22-dehydrocholesterol. Only a few Rhodophyta contain traces of C-28 and C-29 sterols.[12]

Among the isolated compounds, oleic acid is a monounsaturated fatty acid (C-18) found naturally in many plant sources, well known as the omega-9 fatty acid, and considered one of the healthiest sources of fat in the diet (lower risk of coronary heart disease). There are many known plant sources of high-oleic acid oils such as olive, canola, sunflower, safflower, and *Moringa oleifera*.[14] Oleic acid may have protective effects against cardiovascular complications of diabetes since glutathione (GSH), total lipid, and triacylglycerol (TAG) levels are beneficially affected. The decreased tissue factor (TF) activity in diabetic-hyperlipidemic persons may protect these tissues from the risk of thrombosis.[15]

This is the first time that we report the presence of cholesteryl oleate in *H. flagelliformis*. As already reported in the literature, intravenously administered cholesterol oleate emulsion is known to be localized mainly in the Kupffer cells and in the splenic red pulp macrophages. Cultured macrophages treated with this lipid show inhibition of antigen-binding and depressed phagocytosis of heterologous erythrocytes. The lipid does not affect lymphocytes.[16] Among the isolated sterols, (22*E*)-cholesta-5,22-dien-3β-ol-7-one is separated from *H. flagelliformis* for the first time. The effects of dietary algae and cholesterol supplementation on postprandial lipemia and lipoproteinaemia and arylesterase (AE) activity in growing male Wistar rats were also reported.[17]

In conclusion, most of the red algae (and other seaweed) are edible, for example, *Hypnea* used as a salad or in soups. Also, red algae are enriched with cholesterol polysaccharides. Since, high postprandial lipemia increases cardiovascular risk, alga consumption may affect postprandial lipoproteinemia due to its high cholesterol contents.
